# Supplementary material for: Lessons learned from the COVID-19 pandemic about sample access for research in the UK
Source: BMJ Open. 2022 Apr 15;12(4):e047309. doi: 10.1136/bmjopen-2020-047309 (PMC9013782; doi:10.1136/bmjopen-2020-047309)
Supplement: Author's manuscript [file bmjopen-2020-047309.draft_revisions.pdf]

BMJ Open

BMJ Open is committed to open peer review. As part of this commitment we make the peer review history of every article we publish publicly available.

When an article is published we post the peer reviewers' comments and the authors' responses online. We also post the versions of the paper that were used during peer review. These are the versions that the peer review comments apply to.

The versions of the paper that follow are the versions that were submitted during the peer review process. They are not the versions of record or the final published versions. They should not be cited or distributed as the published version of this manuscript.

BMJ Open is an open access journal and the full, final, typeset and author-corrected version of record of the manuscript is available on our site with no access controls, subscription charges or pay-per-view fees (http://bmjopen.bmj.com).

If you have any questions on BMJ Open's open peer review process please email info.bmjopen@bmj.com

BMJ Open

Lessons learned from the COVID- 19 pandemic about sample access

Journal:	*BMJ Open*
Manuscript ID	bmjopen-2020-047309.R1
Article Type:	Communication
Date Submitted by the Author:	23-Jul-2021
Complete List of Authors:	Sims, Jessica; UCL, Surgery and interventional Science<br>Lawrence, Emma; UCL, Surgery and interventional science<br>Glazer, Katy; Kingston University<br>Gander, Amir; UCL, Surgery and interventional Science<br>Fuller, Barry; University College London, Surgery and interventional Science<br>Garibaldi, Jonathan; University of Nottingham, School of Computer Science<br>Davidson, Brian; Royal Free London NHS Foundation Trust, HPB Surgery<br>Quinlan, Philip; University of Nottingham, Advanced Data Analysis Centre (ADAC); University of Nottingham, Health Data Research UK
**Primary Subject Heading**:	Communication
Secondary Subject Heading:	Ethics, Health informatics, Health policy, Health services research
Keywords:	Health informatics < BIOTECHNOLOGY & BIOINFORMATICS, COVID-19, ETHICS (see Medical Ethics), Health policy < HEALTH SERVICES ADMINISTRATION & MANAGEMENT

SCHOLARONE™
Manuscripts

BMJ

I, the Submitting Author has the right to grant and does grant on behalf of all authors of the Work (as defined in the below author licence), an exclusive licence and/or a non-exclusive licence for contributions from authors who are: i) UK Crown employees; ii) where BMJ has agreed a CC-BY licence shall apply, and/or iii) in accordance with the terms applicable for US Federal Government officers or employees acting as part of their official duties; on a worldwide, perpetual, irrevocable, royalty-free basis to BMJ Publishing Group Ltd ("BMJ") its licensees and where the relevant Journal is co-owned by BMJ to the co-owners of the Journal, to publish the Work in BMJ Open and any other BMJ products and to exploit all rights, as set out in our licence.

The Submitting Author accepts and understands that any supply made under these terms is made by BMJ to the Submitting Author unless you are acting as an employee on behalf of your employer or a postgraduate student of an affiliated institution which is paying any applicable article publishing charge ("APC") for Open Access articles. Where the Submitting Author wishes to make the Work available on an Open Access basis (and intends to pay the relevant APC), the terms of reuse of such Open Access shall be governed by a Creative Commons licence – details of these licences and which Creative Commons licence will apply to this Work are set out in our licence referred to above.

Other than as permitted in any relevant BMJ Author's Self Archiving Policies, I confirm this Work has not been accepted for publication elsewhere, is not being considered for publication elsewhere and does not duplicate material already published. I confirm all authors consent to publication of this Work and authorise the granting of this licence.

For peer review only

Note from the Editors: Instructions for reviewers of communication articles

The communication article is a new article type considered by BMJ Open for submissions that do not meet BMJ Open's criteria for a research article but are of interest and relevance to the journal's wide, international readership. The content can be any area of medical research that directly addresses patient outcomes or the practice and delivery of healthcare. Examples include: explanation and elaboration of research reporting guidelines; initiatives aiming to improve data sharing practices; brief reports on the development and evaluation of complex interventions, and; initiatives to reduce research waste and identify priority areas for medical research.

When reviewing this manuscript please consider the following points:

- Are the issues raised by the article important to BMJ Open's broad and international readership that includes patients, researchers, policy makers, health professionals, and doctors of all disciplines?
- Is the article interesting and offering novel insights that have not been sufficiently considered in the existing published literature?
- Is the article well written and is the content clearly presented? Does it have a clear message?
- Will the article help medical researchers, patients or related groups of readers to make better decisions?
- Does the article demonstrate one or more of the following values: transparency, openness, collaboration, innovation, reproducibility, patient/ public involvement, improving peer review and journal best practice, and reducing research waste?

Some additional things to keep in mind when reviewing communication articles:

- Unfortunately, we are unable to customize the reviewer report form for communication articles. As such, most of the items on the form should be scored as Not Applicable (N/A).
- BMJ Open will occasionally consider commentaries, opinion pieces and debates submitted under this new article type.
- Communication articles can include methods, results and discussion sections, if applicable, but these sections are not compulsory.

For peer review only

Lessons learned from the COVID- 19 pandemic **about sample access**

Paper Authors

Jessica Mai Sims1 Emma Lawrence1, Katy Glazer3, Amir Gander1,2, Barry Fuller1,2, Jonathan Garibaldi4,5 and Brian Davidson1,2 Philip R. Quinlan4,5.

Paper Author Affiliations

1. Division of Surgery and Interventional science, Royal Free Campus, UCL, London, UK
2. Tissue Access for Patient Benefit, UCL, London UK
3. Kingston University London, London, UK
4. School of Computer Science, University of Nottingham, Nottingham, UK.
5. Advanced Data Analysis Centre, The University of Nottingham, Nottingham, UK.

Corresponding Author: Dr Emma Lawrence e.lawrence@ucl.ac.uk 020 8016 8103

Division of Surgery and Interventional science, Royal Free Campus, UCL, London, UK.

For peer review only

Abstract

Objective
Annotated clinical samples taken from patients are a foundation of translational medical research and give mechanistic insight in drug trials. Prior research by the Tissue Directory and Coordination Centre (TDCC) indicated that researchers, particularly those in industry, face many barriers in accessing patient samples. As a national coordination centre, the TDCC is tasked with improving efficiency in the biobanking sector. We sought to identify and coordinate UK biobanks able to collect COVID-19 related samples for COVID researchers between March and September 2020.

Findings
Almost a third of UK biobanks were closed during the first wave of the UK COVID-19 pandemic. Of the remainder, 43% had limited capabilities whilst 26% maintained normal activity. Of the nationally prioritised COVID interventional studies, just 3 of the 5 that responded to questioning were collecting human samples. Of the 41 requests for COVID samples received by the TDCC only four could be fulfilled due to a lack of UK coordinated strategy. Meanwhile the reports remain that sample collections in the UK going largely unused.

Conclusion
The response to a pandemic demands high level co-ordinated research responses to reduce mortality. This work highlights the lack of efficiency and coordination between human sample collections and clinical trials in the UK. UK sample access is not working for researchers, clinicians, or patients. A radical change is required in the system for sample collection and distribution to maximise this valuable resource of human donated samples.

For peer review only

Background

Accessing well annotated human samples for medical research is a crucial, yet complex process. Researchers face numerous barriers when it comes to accessing and using human samples, the ethical approval process being one of them [1]. Research has also shown that industry end-users face significant challenges in locating samples for research resulting in the use of inferior alternatives such as animal models [2]. The current system of sample collection is still funded by research project funding that requires and encourages researchers to invest time and resource in the design, ethical and legal governance application processes and the acquisition of human samples. The UKCRC Tissue Directory and Coordination Centre (TDCC) was established in 2014 to publicise existing sample collections and to improve sample access [3]. As of April 2020, the UKCRC TDCC's platform for sample discovery, the Tissue Directory, had 220 human biobanking organisations registered, including biobanks, cohort studies and clinical trials. Researchers can search this freely-accessible, online directory to locate suitable research samples [4]. The TDCC seeks to improve efficiency in the sector by bringing visibility to existing infrastructure. The COVID-19 pandemic provided a challenge to biobanking for biomedical research but also an opportunity to gather data on failures and achievements to better integrate the sector in the future.

A recent survey of human tissue researchers demonstrated that it is still very common practice for researchers to collect samples by established local networks rather than sourcing and utilising samples located in other institutions [1]. Given the time and effort required to secure funding for research, the samples and associated data collected during any specific research programme are often perceived as an asset of the research group (academic or commercial) rather than a resource to be shared for the greater societal good. Therefore, despite the many mechanisms put in place by funders and regulators to promote transparency and re-use of existing infrastructures in research and dissemination of results, there are many understandable reasons and incentives why researchers would be reluctant to share samples widely.

Over a period of 25 weeks during the first wave of the COVID-19 pandemic in the UK (March to September), the TDCC received 41 requests for help to find COVID-19 related samples. The TDCC was able to assist just four researchers to source the samples they required.

Human sample collection and sharing during the COVID pandemic.

All 220 tissue collection organisations registered with the UKCRC Tissue Directory were emailed up to three times enquiring about the state of their operations during the COVID-19 pandemic. One hundred and fifty tissue collection organisations responded (68%). From those, 111, or 74% were closed (68) or had a reduced capacity to operate (43). Only 26 out of 150 organisations (17%) were operating as before the pandemic. The remaining 13 had an unknown operating status. The closures or reduced capacity were categorised as due to:

- Site closure;
- Reallocation of staff or resources to support NHS COVID-19 efforts;
- Health and safety concerns;
- Management or organisational decision;
- Staff working from home; and
- Suspension of non-essential research.

Of the 69 that were functioning in some capacity, just 12 responded that they were able to access COVID-19 patients to collect COVID-19 samples. Further, nine of these (75%) were already supplying

For peer review only

samples to internal projects and were thus unable to share more widely to supply samples to additional external research projects (figure 1).

It is striking that 74% of the UK human tissue collection organisations were either closed or not fully operational during this international health crisis. Nearly half of those that responded to our call were closed completely, rather than being seen to have skills and experience to benefit research or patient care. Also considering the high demand for aid to research into COVID-19, projects and biobanks collecting COVID-19 samples missed an opportunity to support other research through publicising their capacity to collect or supply samples.

Discussion

This problem with sample sourcing impacted the ability of UK Small to Medium Enterprises (SMEs) and public health institutions to access samples for vital research projects [5, 6]. For example, nearly two-thirds of the sample requests to the TDCC came from private research organisations. However, as the remaining third came from university research groups, sample access is a challenge faced by both public and commercial researchers. Current research culture (funders, regulators, rewards, precedent) is not configured to encourage or facilitate sharing, but rather it incentivises competition.

Substantial changes to the system will be required to offer a solution for improved human tissue access [1, 7] . The COVID-19 pandemic has laid bare the consequences of a lack of proactive and upfront coordination and national infrastructure. We need a new strategy for human sample research which will reassure patients that the scientific and biobanking communities are fully utilising their donated samples [8].

Contrast to coordinating and sharing of data

Each nation of the United Kingdom has support services to collect data for the NHS, which becomes a uniquely valuable resource to healthcare researchers on a scale which is unparalleled globally. NHS-X has recently been established to further the utilisation of data in leading data science initiatives. There is no equivalent of NHS-D/X for tissue samples collected in the NHS.

Health Data Research UK (HDR UK) was established in 2018 and has now initiated many high-profile data coordinating efforts with a focus on open standards and interoperability, as the cornerstone of those activities. HDR UK could be considered, in some respects, an equivalent in data as TDCC is for samples. However, the most fundamental difference is that HDR UK has been able to fund theme-based data collection efforts in eight networks (BREATHE, DATA-CAN, Gut Reaction, INSIGHT, NHS DigiTrials, PIONEER, Discover-NOW, BHF Data Science Centre) and general capabilities under national research themes from the original substantive sites (Cambridge, London, Midlands, North, Oxford, Scotland, South-West, Wales and Northern Ireland). HDR UK therefore, has a ready-made funded network in which it can direct and inform change, rather than simply advocating on the benefits of data sharing and collaborative working that has been the historical case for human sample coordination efforts. Although system wide change is a challenge in data, HDR UK and the networks it coordinates, offers a beacon of light for a new way of operating.

The collection of human samples is in many ways a means to an end. Samples are the raw material from which many important clinical problems are investigated and addressed. High quality data is the

For peer review only

biproduct of a high-quality sample pipeline. TDCC has supported a data centric focus on biobanking which places the emphasis on datasets with samples, rather than collections of 'samples with annotated data'. However, this has been an advocacy role that does not have regulatory or funding backing which makes change hard to achieve using this strategy and relies on a collation of the willing.

In response to COVID-19, these data assets were brought to the fore and also further enabled by legislation [9]. £8.2M was invested in a new national data connectivity infrastructure under the National Core Studies (NCS) programme for an initial period of six months that built upon the established funding streams to support HDR UK and the national trusted research environments. This will result in legacy for future generations and data linkage and data infrastructure initiated in response to COVID-19 should easily become the new normal.

In contrast, there was no UK initiative to fund or drive the uplift of coordination efforts for human sample collection and sharing during the pandemic and there was no regulatory change to support better use of samples in response to COVID-19. There were clearly national efforts with a research focus that had a direct or indirect aim of collecting samples, such as COG-UK, ISARIC, and REACT. However, there was no coordination between these studies. This research focus on sample collection, rather than routine, is the likely cause for so many biobanks closing during COVID-19. Instead, they could have been recognised as infrastructure that could be re-purposed as part of the response. If the biobanks are so heavily defined by the research it can support, then it would be a logical consequence that whilst much other research was suspended the resulting biobanks would also be suspended. This had the result that the more service orientated biobanks that were still operational could not respond to the volume of demand than they then experienced.

A national approach to human tissue could bring the same efficiency and high-level governance that has been achieved with NHS data collection. The legal basis for tissue consent and its pathway, (e.g. registration, consent, opt-out; etc.) could all be defined at a national level. The NHS stores millions of human tissue samples each year which could be aligned with national datasets, for both academic and commercial research benefit. Tissue samples are vital to supporting research addressing and preventing diseases of today and tackling the pandemics of tomorrow.

To the future

COVID-19 research has been strongly supported by the public. We have seen record participant recruitment in the RECOVERY trial [10], public registering to be participants to test the new vaccinations, and mass public activities such as the ZOE app, which helped guide symptom advice. The enthusiasm and support from the public could be due to collective effort to support research to tackle an issue affecting everyone, as well as timely information on how their participation is making a difference. The public are equally supportive of biobanking activities and consenting rates are always reported to be high.

Patients may understandably consider that tissue samples donated throughout their life could be optimally used in multiple clinical trials or research studies through distribution from a suitably regulated biobank. Currently, their donated samples remain largely locked within individual studies and samples cannot contribute to national requirements as highlighted by the COVID pandemic. The current approach of funding sample collections within research projects runs the risk that they become defined by that research project. The governance, the consent, the access and the focus are all defined by the study, rather than as a resource to be accessed and reused more widely beyond the local level and a specific project. The consequence is clear to see from COVID-19 where much of the infrastructure was not operational because of the inseparable bond between the research the

For peer review only

biobanks support and the operation of the biobank. The approach taken in COVID-19 repeated previous methods, which is to fund new collection of samples as part of research studies with the consequence of research silos and the only coordination possible being retrospective. To recognise the enormous value of human tissue in research, particularly when linked with national datasets, we must consider new ways of coordination in sample collection activity if we truly seek a different research culture in which samples are not preserved as property of a study but a donated gift from the public to further research.

As an example, what if sample coordination was embedded in relevant initiatives such as the clinical trials undertaken during COVID-19. With a different level of coordination, the RECOVERY trial could have taken a small aliquot of blood or consented for surplus blood to be collected alongside that taken for routine hospital tests? If so, requests for help to access samples received by TDCC could have easily been fulfilled. Thus, supporting a rapid and significant increase in UK research and development activity into COVID-19. This is of course not a criticism of RECOVERY, as they operated within the current constraints and expectations, but aligning tissue or sample collection in parallel could have been achieved without added complexity or delays in the trial planning. In the bigger picture of the overall response to COVID-19 it was a missed opportunity. The challenge is that without a novel and coherent strategy, a coordinated approach for reuse is not seen as an opportunity, but as a radical and unworkable aspiration.

Conclusion

The COVID-19 pandemic revealed that biobanking was not prioritised as an integral service, or infrastructure in its own right, to support research. This outlook can be attributed to a wider lack of national strategy and coordination to facilitate access and efficient use, that is not seen in allied areas of vital research imperative such as health data collection and analysis. Wider sample demand was unmet, despite some facilities actively collecting samples for generic collections and specific studies - some with record participation in research. This indicates missed opportunities to maximise efficient use of resources and raises concern on how donated samples are used. A national strategy for biobanking, backed and reinforced by the governments and large research funders, would ensure a new level of coordination that regards routine sample collection having the same value as routine data collection. This would then ensure each donation could be fully utilised for active research rather than held as an asset for possible future use.

Contributorship statement

JS and EL contributed equally to this paper.

JS, EL, KG, PQ: Substantial contributions to the conception or design of the work; or the acquisition, analysis, or interpretation of data for the work.

ALL: Drafting the work or revising it critically for important intellectual content.

ALL: Final approval of the version to be published.

ALL: Agreement to be accountable for all aspects of the work in ensuring that questions related to the accuracy or integrity of any part of the work are appropriately investigated and resolved.

For peer review only

Competing Interest

There are no competing interests for any author.

Funding

This work was supported by Medical research Council grant number MR/R022488/1.

Data sharing

No additional data available.

References

1. Lawrence, E., et al., *The Barriers and Motivators to Using Human Tissues for Research: The Views of UK-Based Biomedical Researchers.* Biopreserv Biobank, 2020. **18**(4): p. 266-273.
2. The BioIndustry Association and The Medicines Discovery Catapult: *State of the Discovery Nation 2019*. 2019.
3. Quinlan, P.R., Lawrence, E., Pourabdolla, A., Sims, J.M., Gander, A., Fuller, B., Thomson, B.J., Garibaldi, J.M. and Davidson, B, *The UK Clinical Research Collaboration (UKCRC) Tissue Directory and Coordination Centre: The UK's Centre for facilitating the Usage of Human Samples for Medical Research.* Open Journal of Bioresources, 2017.
4. Quinlan, P. *UKCRC Tissue Directory* 2016 [cited 2020 14/12/2020]; Available from: https://www.biobankinguk.org/directory/.
5. Sample, I., *Blood sample shortage holding up UK work on antibody tests*, in *The Guardian*. 2020: The Guardian
6. Ledford, H., *Autopsy slowdown hinders quest to determine how coronavirus kills.* Nature, 2020.
7. NC3Rs, *The use of human tissue in asthma research:* https://www.nc3rs.org.uk/sites/default/files/documents/Human_Tissue_hub/Survey%20report%20-%20Human%20tissue%20use%20in%20asthma.pdf.
8. Medicines Discovery Catapult, I.H., Use My Data, *The Issue with Tissue*. 2018: https://md.catapult.org.uk/resources/the-issue-with-tissue/.
9. COPI Notice to NHS England: https://www.england.nhs.uk/wp-content/uploads/2020/06/COPI-notice-to-nhs-england-improvement-covid-19.pdf March 2020.
10. NIHR. *The UK's flagship COVID-19 treatment trial - the RECOVERY trial*. 2020 [cited 2020 December]; Available from: https://www.nihr.ac.uk/case-studies/the-uks-flagship-covid-19-treatment-trial-the-recovery-trial/25358.

Figure 1: Status of tissue banks in the UK during COVID-19 Pandemic (March – September 2020)

For peer review only

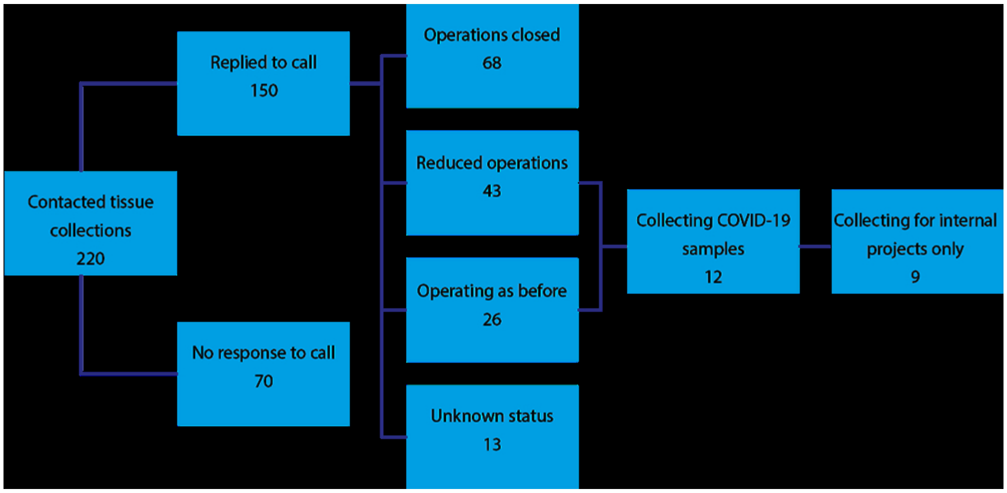

Figure 1: Status of tissue banks in the UK during COVID-19 Pandemic (March – September 2020)

321x155mm (87 x 87 DPI)

Lessons learned from the COVID-19 pandemic about sample access for research in the UK

Journal:	*BMJ Open*
Manuscript ID	bmjopen-2020-047309.R2
Article Type:	Communication
Date Submitted by the Author:	21-Jan-2022
Complete List of Authors:	Sims, Jessica; UCL, Surgery and interventional Science<br>Lawrence, Emma; UCL, Surgery and interventional science<br>Glazer, Katy; Kingston University<br>Gander, Amir; UCL, Surgery and interventional Science<br>Fuller, Barry; University College London, Surgery and interventional Science<br>Davidson, Brian; Royal Free London NHS Foundation Trust, HPB Surgery<br>Garibaldi, Jonathan; University of Nottingham, School of Computer Science<br>Quinlan, Philip; University of Nottingham, Digital Health, School of Medicine
**Primary Subject Heading**:	Communication
Secondary Subject Heading:	Ethics, Health informatics, Health policy, Health services research
Keywords:	Health informatics < BIOTECHNOLOGY & BIOINFORMATICS, COVID-19, ETHICS (see Medical Ethics), Health policy < HEALTH SERVICES ADMINISTRATION & MANAGEMENT

I, the Submitting Author has the right to grant and does grant on behalf of all authors of the Work (as defined in the below author licence), an exclusive licence and/or a non-exclusive licence for contributions from authors who are: i) UK Crown employees; ii) where BMJ has agreed a CC-BY licence shall apply, and/or iii) in accordance with the terms applicable for US Federal Government officers or employees acting as part of their official duties; on a worldwide, perpetual, irrevocable, royalty-free basis to BMJ Publishing Group Ltd ("BMJ") its licensees and where the relevant Journal is co-owned by BMJ to the co-owners of the Journal, to publish the Work in BMJ Open and any other BMJ products and to exploit all rights, as set out in our licence.

The Submitting Author accepts and understands that any supply made under these terms is made by BMJ to the Submitting Author unless you are acting as an employee on behalf of your employer or a postgraduate student of an affiliated institution which is paying any applicable article publishing charge ("APC") for Open Access articles. Where the Submitting Author wishes to make the Work available on an Open Access basis (and intends to pay the relevant APC), the terms of reuse of such Open Access shall be governed by a Creative Commons licence – details of these licences and which Creative Commons licence will apply to this Work are set out in our licence referred to above.

Other than as permitted in any relevant BMJ Author's Self Archiving Policies, I confirm this Work has not been accepted for publication elsewhere, is not being considered for publication elsewhere and does not duplicate material already published. I confirm all authors consent to publication of this Work and authorise the granting of this licence.

For peer review only

Lessons learned from the COVID- 19 pandemic **about sample access for research in the UK**

Paper Authors

Jessica Mai Sims[1], Emma Lawrence[1], Katy Glazer[1,2], Amir Gander[1,3], Barry Fuller[1,3], Jonathan Garibaldi[4], Brian Davidson[1,3] and Philip R. Quinlan[5]

Paper Author Affiliations

1. Division of Surgery and Interventional science, Royal Free Campus, UCL, London, UK
2. Kingston University London, London, UK
3. Tissue Access for Patient Benefit, UCL, London UK.
4. School of Computer Science, University of Nottingham, Nottingham, UK.
5. Digital Health, School of Medicine, The University of Nottingham, Nottingham, UK.

Corresponding Author: Dr Philip Quinlan Philip.Quinlan@nottingham.ac.uk

Abstract

Objective

Annotated clinical samples taken from patients are a foundation of translational medical research and give mechanistic insight in drug trials. Prior research by the Tissue Directory and Coordination Centre (TDCC) indicated that researchers, particularly those in industry, face many barriers in accessing patient samples. The arrival of the COVID-19 pandemic to the UK produced an immediate and extreme shockwave which impacted on the ability to undertake all crucial translational research. As a national coordination centre, the TDCC is tasked with improving efficiency in the biobanking sector. Thus, we took responsibility to identify and coordinate UK tissue sample collection organisations (biobanks) able to collect COVID-19 related samples for researchers between March and September 2020.

Findings

Almost a third of UK biobanks were closed during the first wave of the UK COVID-19 pandemic. Of the remainder, 43% had limited capabilities whilst 26% maintained normal activity. Of the nationally prioritised COVID-19 interventional studies, just three of the five that responded to questioning were collecting human samples. Of the 41 requests for COVID-19 samples received by the TDCC, only four could be fulfilled due to a lack of UK coordinated strategy. Meanwhile in the background there are numerous reports that sample collections in the UK remain largely underutilised.

Conclusion

The response to a pandemic demands high level co-ordinated research responses to reduce mortality. Our study highlights the lack of efficiency and coordination between human sample collections and clinical trials across the UK. UK sample access is not working for researchers, clinicians, or patients. A radical change is required in the strategy for sample collection and distribution to maximise this valuable resource of human donated samples.

For peer review only

For peer review only - http://bmjopen.bmj.com/site/about/guidelines.xhtml

Background

Accessing well annotated human samples for medical research is a crucial, yet complex process. Researchers face numerous barriers when it comes to accessing and using human samples, the ethical approval process being one of them [1]. Research has also shown that industry end-users face significant challenges in locating samples for research resulting in the use of inferior alternatives such as animal models [2]. The current system of sample collection is still supported by research project funding that requires and encourages researchers to invest time and resource in the design, ethical and legal governance application processes and the acquisition of human samples. This is done in competition with other researchers, leading to an inescapable sense of 'ownership' rather than collaboration in the broader scientific endeavour. The UKCRC Tissue Directory and Coordination Centre (TDCC) was established in 2014 to publicise existing sample collections and to improve sample access [3]. As of April 2020, the UKCRC TDCC's platform for sample discovery, the Tissue Directory, had 220 human biobanking organisations registered, including biobanks, cohort studies and clinical trials. Most of these organisations are hosted by public universities or NHS hospitals. Researchers can search this freely-accessible, online directory to locate suitable research samples [4]. The TDCC seeks to improve efficiency in the sector by bringing visibility to existing infrastructure. The COVID-19 pandemic provided a challenge to biobanking for biomedical research but also an opportunity to gather data on failures and achievements to better integrate the sector in the future.

A recent survey of human tissue researchers demonstrated that it is still very common practice for researchers to collect samples by established local networks rather than sourcing and utilising samples located in other institutions [1]. Given the time and effort required to secure funding for research, the samples and associated data collected during any specific research programme are often perceived as an asset of the research group (academic or commercial) rather than a resource to be shared for the greater societal good. Therefore, despite the many mechanisms put in place by funders and regulators to promote transparency and re-use of existing infrastructures in research and dissemination of results, there are many understandable reasons and incentives why researchers would be reluctant to share samples widely.

Over a period of 25 weeks during the first wave of the COVID-19 pandemic in the UK (March to September), the TDCC received 41 requests for help to find COVID-19 related samples. The TDCC was able to assist just four researchers to source the samples they required.

Human sample collection and sharing during the COVID-19 pandemic.

All 220 tissue collection organisations registered with the UKCRC Tissue Directory were emailed up to three times enquiring about the state of their operations during the COVID-19 pandemic. Figure 1 summarises the flow of responses. One hundred and fifty tissue collection organisations responded (68%). From those, 111, or 74% were closed (68) or had a reduced capacity to operate (43). Only 26 out of 150 organisations (17%) were operating as before the pandemic. The remaining 13 had an unknown operating status.

The closures or reduced capacity were categorised as due to:

- Site closure (17, 15.3%).
- Reallocation of staff or resources to support NHS COVID-19 efforts (17; 15.3%).
- Staff working from home or relocated (15; 13.5%).

For peer review only - http://bmjopen.bmj.com/site/about/guidelines.xhtml

- Suspension of non-essential research (10; 9.9%).
- Management or organisational decision (6; 5.4%).
- Study completed or not yet started (2; 1.8%).
- Health and safety concerns (1; 0.1%).
- No information given (43; 38.7%).

Of the 69 that were operating as before (26) or at reduced capacity (43), just 12 responded that they were able to access COVID-19 patients to collect COVID-19 samples. Further, nine of these (75%) were already supplying samples to internal projects and were thus unable to share more widely to supply samples to additional external research projects.

It is striking that 74% of the UK human tissue collection organisations were either closed or not fully operational during this international health crisis. Nearly half of those that responded to our call were closed completely, rather than being seen to have skills and experience to benefit research or patient care. Also considering the high demand for aid to research into COVID-19, projects and organisations collecting COVID-19 samples missed an opportunity to support other research through publicising their capacity to collect or supply samples.

Discussion

This problem with sample sourcing impacted the ability of UK Small to Medium Enterprises (SMEs) and public health institutions, and expert academic research groups to access samples for vital research projects [5, 6]. For example, nearly two-thirds of the sample requests to the TDCC came from private research organisations. However, as the remaining third came from university research groups, sample access is a challenge faced by both public and commercial researchers. Current research culture (funders, regulators, rewards, precedent) is not configured to encourage or facilitate sharing, but rather it incentivises competition.

Substantial changes to the system will be required to offer a solution for improved human tissue access [1, 7]. The COVID-19 pandemic has laid bare the consequences of a lack of a national strategy with proactive and upfront coordination and national infrastructure. We need a new approach for human sample research which will reassure patients that the scientific and biobanking communities are fully utilising their donated samples [8] in a manner which is aligned with the ethos of consent given during donation.

Contrast to coordinating and sharing of data

Each nation of the United Kingdom has support services to collect data for the NHS, which becomes a uniquely valuable resource to healthcare researchers on a scale which is unparalleled globally. NHS-X has recently been established to further the utilisation of data in leading data science initiatives. In stark contrast there is no equivalent of NHS-D/X for tissue samples collected in the NHS.

Health Data Research UK (HDR UK) was established in 2018 and has now initiated many high-profile data coordinating efforts with a focus on open standards and interoperability, as the cornerstone of those activities. HDR UK could be considered, in some respects, an equivalent in data as TDCC is for patient samples. However, the most fundamental difference is that HDR UK has been able to fund theme-based data collection efforts in eight networks (BREATHE, DATA-CAN, Gut Reaction, INSIGHT, NHS DigiTrials, PIONEER, Discover-NOW, BHF Data Science Centre) and general capabilities under national research themes from their original dedicated and substantive sites (Cambridge, London, Midlands, North, Oxford, Scotland, South-West, Wales, and Northern Ireland). HDR UK therefore, has

For peer review only - http://bmjopen.bmj.com/site/about/guidelines.xhtml

a ready-made funded network in which it can direct and inform change, rather than simply advocating on the benefits of data sharing and collaborative working which has been the historical and disjointed case for human sample coordination efforts. Although system wide change is a challenge in data, HDR UK and the networks it coordinates, offers a beacon of light for a new way of operating.

The collection of human samples is in many ways a means to an end. Samples are the raw material from which many important clinical problems are investigated and addressed. High quality data is the biproduct of a high-quality sample pipeline. TDCC has supported a data centric focus on biobanking which places the emphasis on datasets with samples, rather than collections of 'samples with annotated data'. However, this has been an advocacy role that does not have regulatory or funding backing which makes change hard to achieve using this strategy and relies on a collation of the willing.

In response to COVID-19, these data assets were brought to the fore and also further enabled by legislation [9]. £8.2M was invested in a new national data connectivity infrastructure under the National Core Studies (NCS) programme for an initial period of six months that built upon the established funding streams to support HDR UK and the national trusted research environments. This will result in legacy for future generations and data linkage and data infrastructure initiated in response to COVID-19 should easily become the new normal.

In contrast, there was no UK initiative to fund or drive the uplift of coordination efforts for human sample collection and sharing during the pandemic, and there was no regulatory change to support better use of samples in response to COVID-19. There were clearly national efforts with a research focus that had a direct or indirect aim of collecting samples, such as COG-UK, ISARIC, and REACT. However, once again there was no coordination between these studies. This research focus on sample collection, rather than sustaining operability, is the likely cause for so many tissue collection organisations closing during COVID-19. Instead, they could have been recognised as infrastructure with technical expertise, regardless of their research specialism, that could be re-purposed as part of a national response. For example, if biobanks are so heavily defined by the research they can support, then it would be a logical consequence that whilst much other research was suspended, their operations would also be suspended. This had the result that the more service orientated biobanks that were still operational could not respond to the volume of demand than they then experienced.

A national approach to human tissue could bring the same efficiency and high-level governance that has been achieved with NHS data collection. The legal basis for tissue consent and its pathway, (e.g. registration, consent, opt-out; etc.) could all be defined at a national level. The NHS stores millions of human tissue samples each year which could be aligned with national datasets, for both academic and commercial research benefit. Tissue samples are vital to supporting research addressing and preventing diseases of today and tackling the pandemics of tomorrow.

To the future

COVID-19 research has been strongly supported by the public. We have seen record participant recruitment in the RECOVERY trial [10], public registering to be participants to test the new vaccines and symptom-alleviating medicines, and mass public activities such as the ZOE app, which helped guide symptom advice. The enthusiasm and support from the public could be due to collective effort to support research to tackle an issue affecting everyone, as well as timely information on how their participation is making a difference. The public are equally supportive of biobanking activities in other spheres and consenting rates are always reported to be high.

Patients may understandably consider that tissue samples donated throughout their life and in altruistic fashion could be optimally used in multiple clinical trials or research studies through

For peer review only - http://bmjopen.bmj.com/site/about/guidelines.xhtml

distribution from a suitably regulated biobank. However, currently their donated samples remain largely locked within individual studies and samples cannot contribute to national requirements as highlighted by the COVID-19 pandemic. The current approach of funding sample collections within research projects runs the risk that they become defined by that research project. The governance, the consent, the access and the focus are all defined by the study, rather than as a resource to be accessed and reused more widely towards the greater societal health benefit. The consequence is clear to see from COVID-19 where much of the infrastructure was not operational because of the inseparable bond between the individual research themes which the biobanks support and the operation of the biobank. The national approach taken in COVID-19 repeated previous methods, which is to fund new collections of samples as part of research studies, with the consequent development of research silos where only retrospective collection was possible. To recognise the enormous value of human tissue in research, particularly when linked with national datasets, we must consider new ways of coordination in sample collection activity if we truly seek a different research culture in which samples are not preserved as property of a study but a donated gift from the public to further research.

As an example, what if sample coordination was embedded in relevant initiatives such as the clinical trials undertaken during COVID-19. With a different level of coordination, the RECOVERY trial could have taken a small aliquot of blood or consented for surplus blood to be collected alongside that taken for routine hospital tests. This then could have fulfilled sample access requests, like those received by TDCC, and have supported a rapid and significant increase in UK research and development activity into COVID-19. This is of course not a criticism of RECOVERY, as they could only operate within the current constraints and expectations. However, aligning tissue or sample collection in parallel could have been achieved without added complexity or delays in the trial planning. In the bigger picture of the overall response to COVID-19 it was a missed opportunity. The challenge is that without a novel and coherent strategy, a coordinated approach for reuse is not seen as an opportunity, but as a radical and unworkable aspiration.

Conclusion

The COVID-19 pandemic revealed that biobanking was not prioritised as an integral service, or infrastructure in its own right, to support crucial frontline research. This picture can be attributed to a wider lack of national strategy and coordination to facilitate access and efficient use of donated patient samples, that is not seen in allied areas of vital research imperative such as health data collection and analysis. Where time was of the essence at the start of the pandemic, wider sample demand was unmet, despite some facilities actively collecting samples for generic collections and specific studies – some with record participation in research. This indicates missed opportunities to maximise efficient use of resources and raises concern on how donated samples are used. A national strategy for biobanking, backed and reinforced by the governments, regulators and large research funders, would ensure a new level of coordination that regards routine sample collection as a national resource having the same value as routine data collection. This would then ensure each donation could be fully utilised for active research rather than held as an asset for possible future use.

Contribution statement

JS and EL contributed equally to this paper.

For peer review only - http://bmjopen.bmj.com/site/about/guidelines.xhtml

JS, EL, KG, PQ: Substantial contributions to the conception or design of the work and the acquisition, analysis, and interpretation of data for the work. They assisted in drafting the work and revising it critically for important intellectual content. All give final approval of the version to be published and agreement to be accountable for all aspects of the work in ensuring that questions related to the accuracy or integrity of any part of the work are appropriately investigated and resolved.

BF, JG, BD made substantial contributions to the analysis and interpretation of data for the work and revising it critically for important intellectual content. All give final approval of the version to be published and agreement to be accountable for all aspects of the work in ensuring that questions related to the accuracy or integrity of any part of the work are appropriately investigated and resolved.

Competing interests

There are no competing interests for any author

Funding

This work was supported by Medical Research Council grant number MR/R022488/1

Data sharing statement

No additional data available.

References

1. Lawrence, E., et al., *The Barriers and Motivators to Using Human Tissues for Research: The Views of UK-Based Biomedical Researchers.* Biopreserv Biobank, 2020. **18**(4): p. 266-273.
2. The BioIndustry Association and The Medicines Discovery Catapult: *State of the Discovery Nation 2019*. 2019.
3. Quinlan, P.R., Lawrence, E., Pourabdolla, A., Sims, J.M., Gander, A., Fuller, B., Thomson, B.J., Garibaldi, J.M. and Davidson, B, *The UK Clinical Research Collaboration (UKCRC) Tissue Directory and Coordination Centre: The UK's Centre for facilitating the Usage of Human Samples for Medical Research.* Open Journal of Bioresources, 2017.
4. Quinlan, P. *UKCRC Tissue Directory* 2016 [cited 2020 14/12/2020]; Available from: https://www.biobankinguk.org/directory/.
5. Sample, I., *Blood sample shortage holding up UK work on antibody tests*, in *The Guardian*. 2020: The Guardian
6. Ledford, H., *Autopsy slowdown hinders quest to determine how coronavirus kills.* Nature, 2020.
7. NC3Rs, *The use of human tissue in asthma research:* https://www.nc3rs.org.uk/sites/default/files/documents/Human_Tissue_hub/Survey%20report%20-%20Human%20tissue%20use%20in%20asthma.pdf.
8. Medicines Discovery Catapult, I.H., Use My Data, *The Issue with Tissue*. 2018: https://md.catapult.org.uk/resources/the-issue-with-tissue/.

For peer review only - http://bmjopen.bmj.com/site/about/guidelines.xhtml

9. COPI Notice to NHS England: https://www.england.nhs.uk/wp-content/uploads/2020/06/COPI-notice-to-nhs-england-improvement-covid-19.pdf March 2020.
10. NIHR. *The UK's flagship COVID-19 treatment trial - the RECOVERY trial*. 2020 [cited 2020 December]; Available from: https://www.nihr.ac.uk/case-studies/the-uks-flagship-covid-19-treatment-trial-the-recovery-trial/25358.

Figure Legends

Figure 1: Status of tissue banks in the UK during COVID-19 Pandemic (March – September 2020)

For peer review only

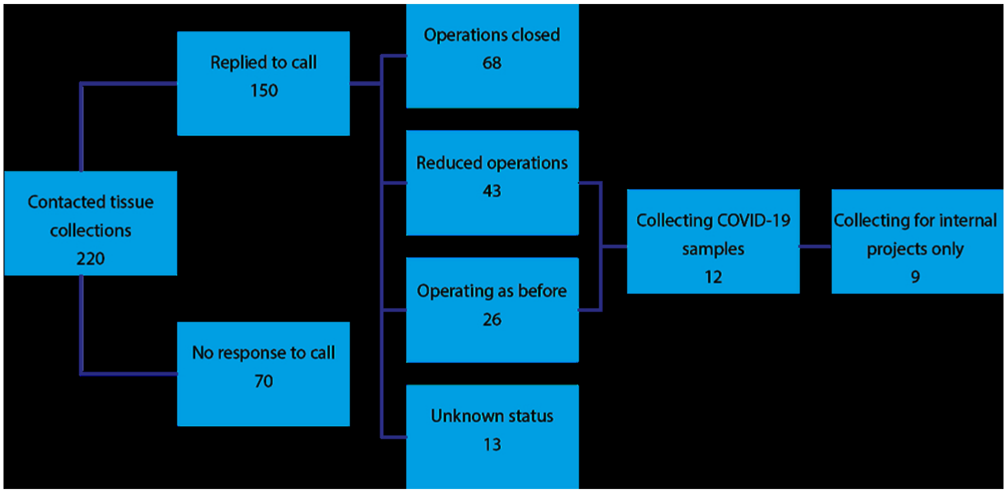

Figure 1: Status of tissue banks in the UK during COVID-19 Pandemic (March – September 2020)

321x155mm (87 x 87 DPI)
